# Dataset on statistical analysis of jet A-1 fuel laboratory properties for on-spec into-plane operations

**DOI:** 10.1016/j.dib.2018.05.083

**Published:** 2018-05-23

**Authors:** Aderibigbe Israel Adekitan, Tobi Shomefun, Temitope M. John, Bukola Adetokun, Alex Aligbe

**Affiliations:** a Department of Electrical and Information Engineering, Covenant University, Ota, Nigeria; b Department of Electrical Engineering, Pan African University Institute for Basic Sciences, Technology and Innovation (PAUSTI), at Jomo Kenyatta University of Agriculture and Technology (JKUAT), Kenya

**Keywords:** Air transportation, Aviation Turbine Kerosene – ATK, Data pattern recognition, Jet A-1 aviation fuel, Jet fuel properties prediction, Quality analysis

## Abstract

Safety is of utmost essence in the aviation sector, both on-ground and in the air. Aviation Turbine Kerosene (ATK) commonly referred to as jet fuel is one of the major resources of the aviation sector, contributing significantly to the operating cost of an airline. Flight safety is a top-notch requirement in air transportation management. Jet fuel quality affects flight safety, and this makes it mandatory to ensure that, at all points in the jet A-1 aviation fuel supply chain, the jet fuel is contamination free and on-spec. Jet fuel quality is determined via various mandatory Joint Inspection Group (JIG) based quality analysis test procedures; both baseline and extensive lab tests by third party labs. Acceptable parameter range has been established for each jet fuel property, the electrical conductivity of jet A-1 fuel must be between 50 and 600 pS/m and the density at 15 °C must be between 0.775 and 0.840 g/cm^3^. Beyond this range, the fuel is deemed off-spec and unsafe for into-plane fuelling operations. This data article presents daily jet fuel test records for jet-A1 fuel. The dataset contains the date of the test, the conductivity, the specific gravity at ambient temperature, the converted specific gravity at 15 °C, and the temperature of the jet fuel sample under study. All the tests were performed at standard laboratory conditions using approved and certified equipment. The dataset provides an opportunity for developing a predictive model that can be used for jet fuel properties prediction on a given day, based on previous data trends and analysis using data pattern recognition, as an indication of the variation of jet fuel properties with daily weather variation.

## Specifications Table

**Table:** 

Subject area	*Engineering*
More specific subject area	*Petrochemical Engineering, Quality Assurance Engineering, Pattern Recognition*
Type of data	*Tables, figures and spread sheet file*
How data was acquired	*Data acquisition from daily, laboratory standard test logs for jet A-1 fuel. The tests were carried out after daily tank draining using chemical water detector, calibrated and certified thermometer and hydrometer, and fuel conductivity meter*
Data format	*Raw, filtered, analyzed*
Experimental factors	*Data was extracted on four (4) jet fuel test parameters, together with the date of the fuel test; from aviation fuel, standard test records of an into-plane company. Only days with four complete test results were considered.*
Experimental features	*Frequency distributions, Linear regression models and Generalized linear model analysis were performed to illustrate data trends, and to determine the relationship among the test data parameters*
Data source location	*Airfield aviation fuel depot based in Nigeria*
Data accessibility	*The dataset is available in a spreadsheet file attached to this article*

## Value of the data

• The dataset presents a detailed Joint Inspection Group (JIG) compliant jet A-1 fuel test results, which shows the variation of jet fuel properties across months in a tropical African country.
• The tables, frequency distribution, and figures presented, provides vital insights on the changes in jet fuel characteristic properties with daily weather variations.
• The data and statistics presented in this data article, with further analysis can be deployed for evolving a very accurate predictive model [1] that is capable of predicting jet fuel properties all through the year. These statistical representations were developed using similar methods to those found in [2].
• Accurate jet fuel properties prediction via data trending analysis, will empower jet fuel depots to proactively prepare sufficiently in terms of quality and procedural requirements to meet any anticipated jet fuel property variation beyond acceptable limits on a given day.
• The availability of this data, will stimulate the collection of similar data for related studies in various regions of the world, and this may trigger further extensive studies and create platforms for collaborative research works on a wider scale, both locally and globally.

## 1 Data

**Fig. 1:**
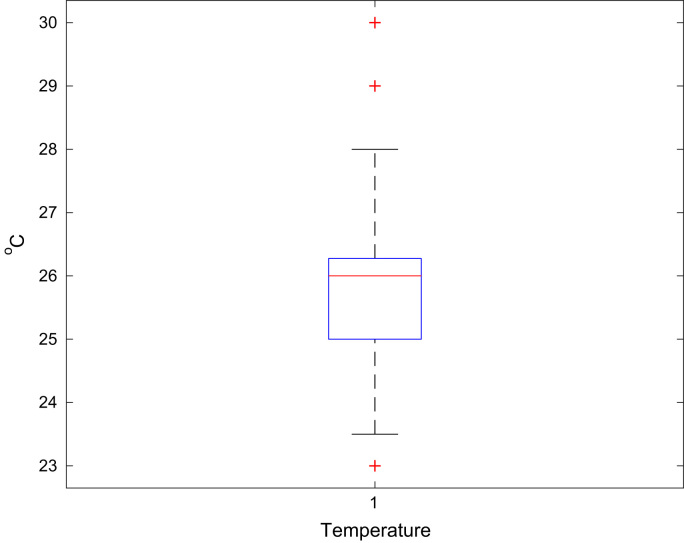
Boxplot of the jet-A1 temperature data set.

**Fig. 2:**
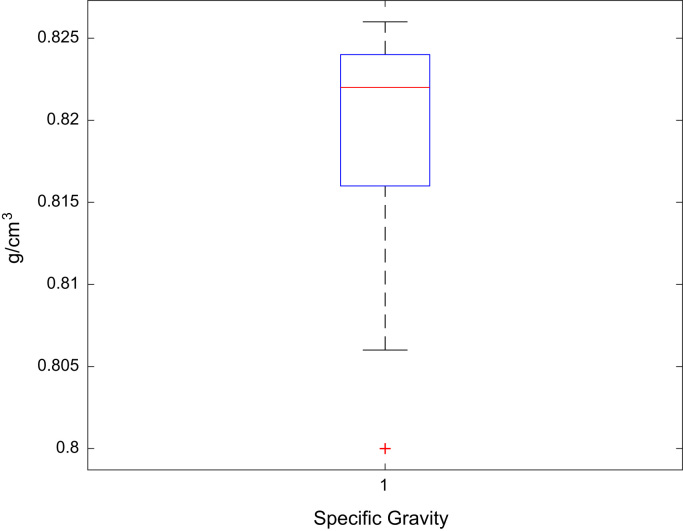
Boxplot of the jet A-1 S.G. data set.

**Fig. 3:**
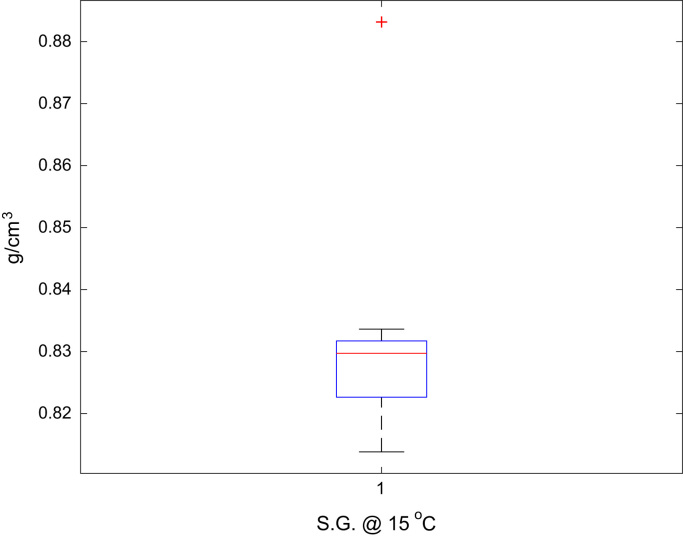
Boxplot of the jet A-1 S.G. @ 15 °C data set.

**Fig. 4:**
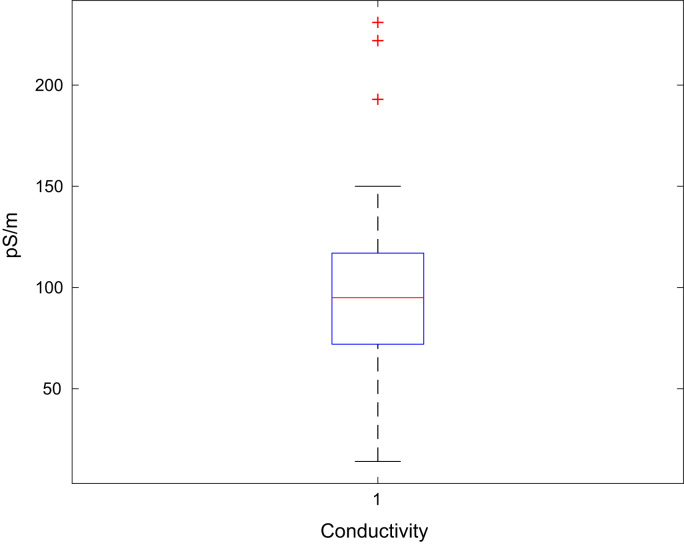
Boxplot of the jet A-1 conductivity data set.

**Fig. 5:**
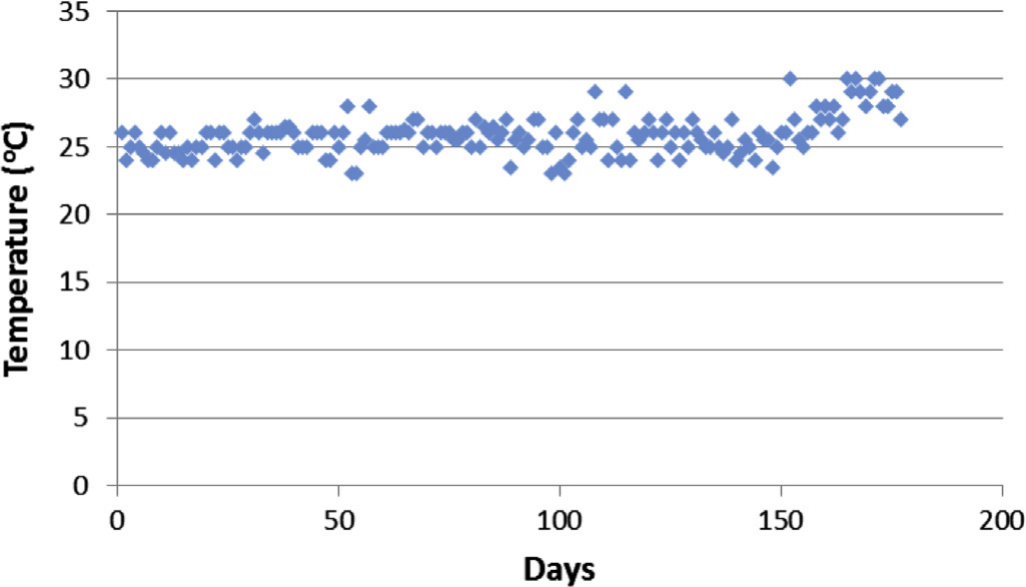
Scatter diagram for the jet fuel temperature dataset.

**Fig. 6:**
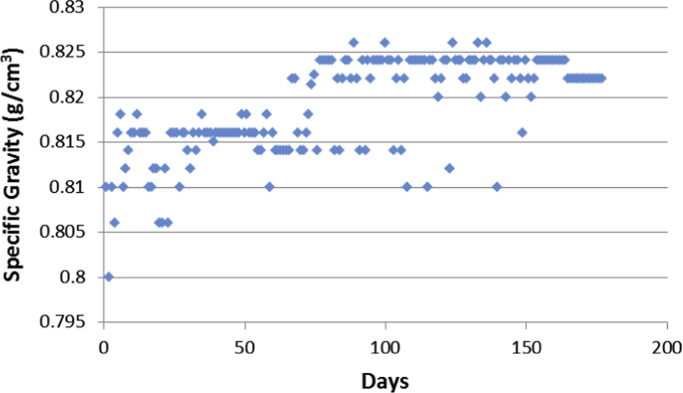
Scatter diagram for the jet fuel S.G. dataset.

**Fig. 7:**
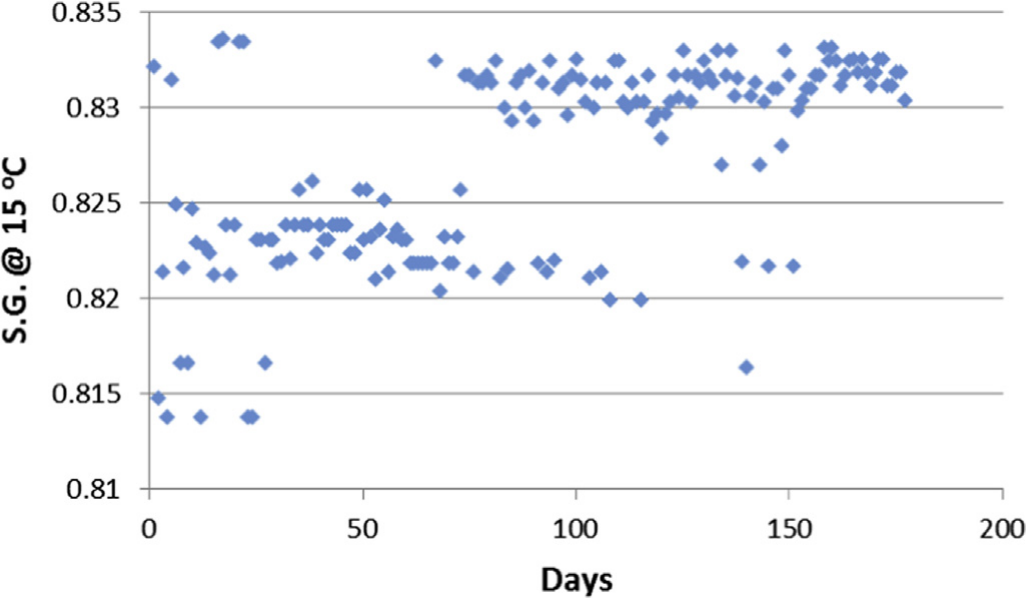
Scatter diagram for the S.G. at 15 °C dataset.

**Fig. 8:**
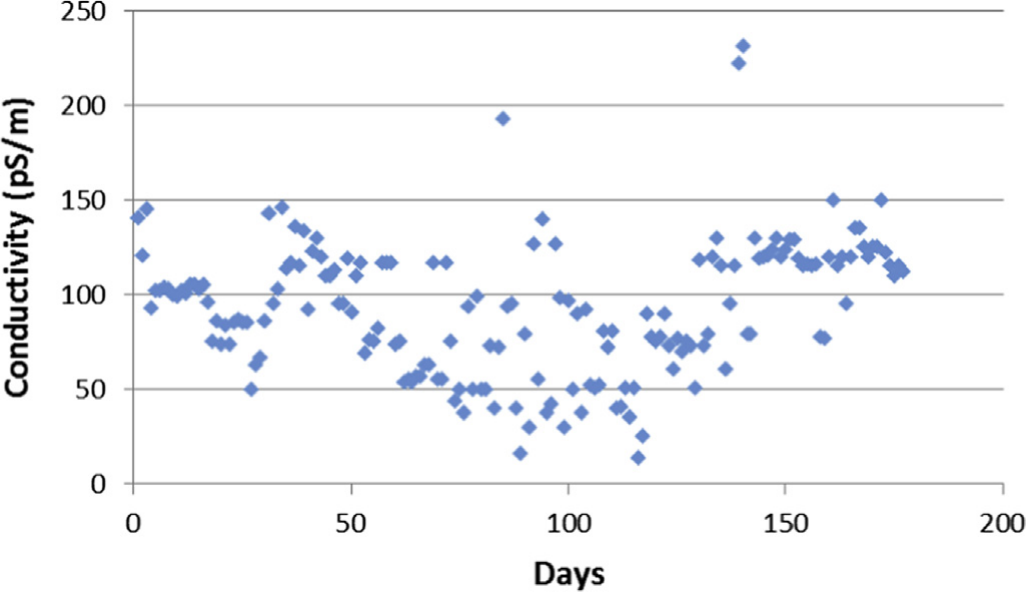
Scatter diagram for the Jet fuel conductivity test data.

Aircrafts runs on aviation fuels, which are majorly of two types; Aviation Gasoline (AVGAS) and Aviation Turbine Kerosene (ATK) [3]. The geographical location of a country determines its weather and climatic conditions, and because of the peculiarities of the properties of jet fuel, the prevailing weather determines the type of jet fuel that is approved for use in each country, in order to prevent freezing at high altitudes. The airlines in Nigerian run on jet-A1 fuel [4]. Jet fuel can be contaminated during transportation [3] and this has been associated with aircraft accidents in the past [5], [6], [7]. Consequently, jet fuel quality and management is one of the determinants of flight safety [8]. The data contained in the attached supplementary spread sheet file, presents mandatory laboratory, daily test records for jet fuel samples subjected to standard JIG test procedures and analysis at an airfield jet fuel depot in Nigeria. The dataset presents 5 key parameters, the date of the lab test, the specific gravity (S.G.) of the jet fuel at ambient temperature, the converted specific gravity of the jet fuel using standard chart at 15 °C (S.G. @ 15 °C), the temperature of the jet fuel (°C) and the conductivity of the jet fuel (pS/m). Table 1, Table 2, Table 3, Table 4, Table 5, Table 6, Table 7, Table 8 present the descriptive statistics of the data and the statistical results of the generalized linear model and linear regression model. The boxplots of the jet fuel parameters are displayed by Fig. 1, Fig. 2, Fig. 3, Fig. 4. The distribution of each data point in the data set is shown by the scatter diagram of Fig. 5, Fig. 6, Fig. 7, Fig. 8.

**Table 1:** Descriptive statistics of jet fuel test parameters.

	**Temperature**	**SG**	**SG @ 15 °C**	**Conductivity**
**Mean**	25.8401	0.8189	0.8273	92.9718
**Sum**	4573.7	144.9448	146.4369	16,456
**Min**	23	0.8	0.8138	14
**Max**	30	0.826	0.8832	231
**Range**	7	0.026	0.0694	217
**Variance**	2.1038	0	0	1210.1412
**Standard Deviation**	1.4504	0.0052	0.0066	34.7871
**Standard Error of Mean**	0.109	0.0004	0.0005	2.6148
**Median**	26	0.822	0.8297	95
**Mode**	26	0.824	0.8317	115.00^a^

a Multiple modes exist, the smallest value is shown.

**Table 2:** Goodness of fit for the generalized linear model.

	**Value**	**d*f***	**Value/d*f***
**Deviance**	1.332	172	0.008
**Scaled Deviance**	177	172	
**Pearson Chi-Square**	1.332	172	0.008
**Scaled Pearson Chi-Square**	177	172	
**Log Likelihood**^b^	181.573		
**Akaike׳s Information Criterion (AIC)**	− 351.147		
**Finite Sample Corrected AIC (AICC)**	− 350.653		
**Bayesian Information Criterion (BIC)**	− 332.09		
**Consistent AIC (CAIC)**	− 326.09		

Dependent Variable: NDATE.

Model: (Intercept), TEMP, SG, CONDUCTIVITY, S.G @ 15 °C^a^.

^a^Information criteria are in smaller-is-better form.

^b^The full log likelihood function is displayed and used in computing.

**Table 3:** Omnibus test.

**Likelihood ratio Chi-square**	**d*f***	**Sig.**
169.111	4	0

Dependent Variable: NDATE.

Model: (Intercept), TEMP, SG, CONDUCTIVITY, S.G @ 15 °C^a^.

^a^Compares the fitted model against the intercept-only model.

**Table 4:** Tests of model effects.

Source	Type III		
	Wald Chi-square	d*f*	Sig.
**(Intercept)**	3,576,374.379	1	0
**Temperature**	38.492	1	0
**SG**	103.917	1	0
**Conductivity**	4.075	1	0.044
**S.G @ 15 °C**	3.072	1	0.08

Dependent Variable: NDATE.

Model: (Intercept), TEMP, SG, CONDUCTIVITY, S.G @ 15 °C.

**Table 5:** Parameter estimates.

		Std. error	95% Wald confidence interval		Hypothesis test		
Parameter	*B*		Lower	Upper	Wald Chi-square	d*f*	Sig.
**(Intercept)**	2000.182	1.0577	1998.109	2002.255	3,576,374.379	1	0
**Temperature**	0.029	0.0047	0.02	0.038	38.492	1	0
**SG**	16.077	1.5771	12.986	19.168	103.917	1	0
**Conductivity**	0	0.0002	1.13E − 05	0.001	4.075	1	0.044
**S.G @ 15 °C**	2.179	1.2431	− 0.258	4.615	3.072	1	0.08
**(Scale)**	0.008^a^	0.0008	0.006	0.009			

Dependent Variable: NDATE.

Model: (Intercept), TEMP, SG, CONDUCTIVITY, S.G @ 15 °C.

a Maximum likelihood estimate.

**Table 6:** Linear regression model summary.

**Model**	***R***	***R* square**	**Adjusted R square**	**Std. error of the estimate**
1	0.784^a^	0.615	0.606	0.087997

a Predictors: (Constant), S.G @ 15 °C, CONDUCTIVITY, TEMP, SG.

**Table 7:** ANOVA.

**Model**	**Sum of squares**	**d*f***	**Mean square**	***F***	**Sig.**
**Regression**	2.131	4	0.533	68.791	0.000^a^
**Residual**	1.332	172	0.008		
**Total**	3.463	176			

a Predictors: (Constant), S.G @ 15 °C, CONDUCTIVITY, TEMP, SG.

**Table 8:** Linear regression model coefficients.

	**Unstandardized coefficients**		**Standardized coefficients**		
**Model**	*B*	Std. error	Beta	*t*	Sig.
**(Constant)**	2000.182	1.073		1864.228	0
**Temperature**	0.029	0.005	0.301	6.116	0
**SG**	16.077	1.6	0.599	10.049	0
**Conductivity**	0	0	0.096	1.99	0.048
**S.G @ 15 °C**	2.179	1.261	0.103	1.728	0.086

## 2 Experimental design, materials and methods

Laboratory tests are performed on samples of jet fuel from each storage tank in a jet fuel depot, and a release certificate must be issued by the laboratory before the operations team can be authorized to pump jet fuel from any storage tank to bowsers or hydrant system for aircraft fuelling. The storage tank is first drained in the morning to remove any water that has settled at the tank base. After draining the water through the flush tank, Jet fuel samples are then taken in visible glass jar and a vortex swirl test is performed to identify particulate matter. The jet fuel must be clear, bright and visually free from solid matter and un-dissolved water [3]. The jet fuel sample is subjected to further tests to determine its conductivity, the specific gravity at ambient temperature, the converted specific gravity at 15 °C, and the temperature of the jet fuel for that day. The values of these measured parameters are recorded in a log for that particular date. The data set was compiled from lab records of daily, JIG based jet A-1 fuel tests. The data set spans a total of 177 days across seven (7) months. Data on the five, key jet fuel parameters were profiled and analysed to identify any hidden relationships among the parameters. Jet fuel properties are significantly influenced by the quality of the handling process and the prevailing weather. Weather varies with seasons and days; hence, jet fuel properties may be predicted for a specific date using known trends. In this data article, the DATE parameter is normalised to generate the NDATE parameter which is an indicator of weather variation on different days. Statistical analysis was carried out to identify hidden relationship between the target NDATE and the predictors; S.G, S.G. @ 15 °C, the temperature of the jet fuel and the conductivity of the jet fuel.
